# The Influence of Marital Status on the Survival of Patients with Uveal Melanoma

**DOI:** 10.1155/2020/7012940

**Published:** 2020-12-04

**Authors:** Wenting Cai, Jiaqi Fan, Tianyi Shen, Jing Yu

**Affiliations:** ^1^Department of Ophthalmology, Shanghai Tenth People's Hospital, Tongji University, School of Medicine, Shanghai, China; ^2^Department of Ophthalmology, Nanjing Medical University, Nanjing, China; ^3^Department of Ophthalmology, Ninghai First Hospital, Ninghai, Zhejiang, China

## Abstract

**Background:**

Uveal melanoma (UM) is the most common primary intraocular tumor in adults and arises from the uvea. Marital status was a vital factor among physical conditions and social networks of cancer patients. Our study aimed to evaluate the impact of marital status on the outcomes among patients with UM.

**Methods:**

Patients with UM newly diagnosed from 2004 to 2015 were extracted, and the data were extracted from Surveillance, Epidemiology, and End Results (SEER) program. Overall survival (OS) was measured via the log-rank test, as well as cancer-specific survival (CSS) was also calculated via the same method. Cox proportional hazards models were applied to assess whether marital status was related to both OS and CSS. Furthermore, we performed subgroup analysis depending on different sexes and SEER stages.

**Results:**

In total, 4217 eligible patients were involved. Of these patients, 66.2% (*n* = 2793) were married, 14.6% (*n* = 615) were single, and 9.0% (*n* = 379) were divorced or separated, as well as widowed were 10.2% (*n* = 430). The 5-year OS of married, single, divorced or separated, and widowed patients was 74.0%, 72.8%, 68.6%, and 55.8%, respectively. The results indicating better OS and CSS occurred among married patients. Other factors such as sex, age at diagnosis, and SEER stage were also correlated with survival in UM patients. Furthermore, subgroup analyses were consistent with the results above.

**Conclusion:**

Marital status was proved to be an independent prognostic value for survival in UM patients. In addition, contrast to married patients, widowed individuals showed poor OS and CSS at different subgroup analyses.

## 1. Introduction

Ocular melanoma is the most common form of primary cancer that influences ocular health [1]. Of these, uveal melanoma (UM) is the most common primary intraocular tumor in adults that arises from the uvea, including the iris, ciliary body, and choroid. The prevalence of UM diagnosed in the United States was 4.9 cases per million every year [2, 3]. Of these cases, 85% originate from the choroidal body, and the remaining originate from the iris and ciliary body. The 5-year overall survival in patients with UM was 76.8%, while the 5-year cancer-specific survival was 84.1% [4]. Previous studies reported that age, histologic type, surgery, and radiotherapy were correlated with prognosis [5]. Considering the prognostic factors associated with UM might provide new potential strategies for UM management and prevention.

Marital status, as a mediating marker of social family condition and mental status, is considered a vital factor that offers modified physical conditions and social networks among patients with a variety of cancers [6–10]. Xie et al. found marital status was closely related with astrocytoma patients, and more physiological support was recommended to patients with aborted marriage [11]. In patients with uterine cancer, the results indicated that poor outcomes were observed in widowed patients, while marriage contributed to a better prognosis, most likely due to spiritual and emotional support [12], yet there seems to be no study investigating the influence of marital status on UM patients. In general, we sought to investigate whether marital status could be a prognostic factor for UM and to explore suitable and timely supporting.

## 2. Materials and Methods

### 2.1. Data Source and Selection Criteria

SEER*∗*Stat software, version 8.3.5, (National Cancer Institute, Bethesda, MD) was used to download primary data.

**Figure 1:**
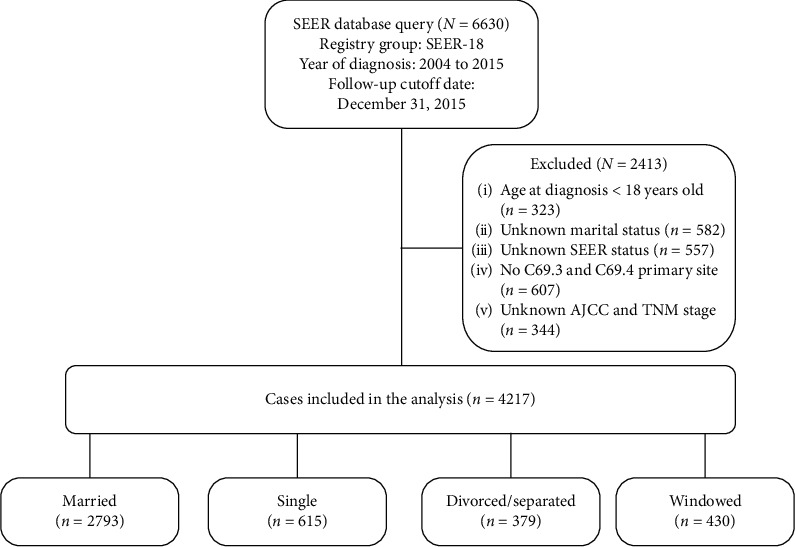
Flowchart describing the inclusion and exclusion of patients.

We extracted UM patients diagnosed between 2004 and 2015 to perform our further analysis. The data regarding sex, marital status, diagnosis year, age at diagnosis, race, SEER stage, histology type, AJCC stage, TNM stage, and treatment information about surgery, as well as median household income, were extracted. A total of 2413 patients were excluded (323 were under 18 years old, 582 were missing marital status information, 557 were missing SEER stage information, 607 were not at C69.3 or 69.4 labeled primary sites, and 344 were missing AJCC and TNM stage information). The patient selection criteria are shown in Figure 1.

### 2.2. Variables and Outcomes

Age was reported in ranges of 18–40, 40–59, 60–79, and ≥80 years, while race was categorized as black, white, others, or unknown using the SEER database. Marital status was categorized as married, single, divorced/separated, and widowed. Data related to diagnosis year (2004–2006, 2007–2009, 2010–2012, 2013–2015), SEER stage (localized, regional, metastatic), histology type (mixed epithelioid and spindle cell melanoma, malignant melanoma, NOS, spindle cell melanoma, others), AJCC stage (I, II, III, IV), and surgery were also obtained. Median household income was classified as <$4006, $4006-$4583, $4583-$5427, or ≥$5427. Registry sites included West (Alaska, California, Hawaii, New Mexico, Utah, Washington), Northeast (Connecticut, New Jersey), Midwest (Iowa, Michigan), and South (Georgia, Kentucky, Louisiana). Overall survival (OS) and cancer-specific survival (CSS) were set as primary end points.

### 2.3. Ethical Approval and Consent

Human participants in the present study were all subject to the ethical standards of the institutional research committee and also 1964 Helsinki Declaration as well as later amendments or comparable ethical standards. No animal studies were included in the present study.

### 2.4. Statistical Analysis

In our study, the relevant factors such as sex, diagnosed age and year, race, SEER stage, histology type, marital status, presence or absence of surgery, AJCC stage, median household income, as well as registry site. Data are presented as mean ± SD. Categorical variables were recorded as counts (percentages). Continuous variables with symmetric distributions across subgroups of marital status were analyzed by variance. OS and CSS served as the primary outcomes.

The Kaplan–Meier method was plotted for assessing survival distributions. Chi-square tests were carried out to assess categorical variables between subgroups. Cox proportional hazards models were performed for assessing therapies for four marital statuses using hazard ratios (HRs) as well as 95% confidence intervals (CIs). SPSS Statistics, version 20, was used to complete all statistical analyses. *P* values lower than 0.05 were evaluated as statistically significant.

## 3. Results

### 3.1. Baseline Clinical Characteristics of UM Patients

In total, 4217 patients with UM were enrolled, including 2215 (52.5%) male and 2002 (47.5%) female individuals, and the median follow-up of all individuals was 45 months (range 0–143 months). Of these individuals, 66.2% (*n* = 2793) were married, 14.6% (*n* = 615) were single, 9.0% (*n* = 379) were divorced or separated, and 10.2% (*n* = 430) were widowed. The 3-year OS in married, single, divorced or separated, and widowed individuals was 83.6%, 83.5%, 82.2%, and 71.1%, respectively. Furthermore, the 5-year OS of patients who were married, single, divorced or separated, and widowed was 74.0%, 72.8%, 68.6%, and 55.8%, respectively. As shown in Table 1, the detailed clinical characteristics of individuals categorized by different forms of marital status were listed.

The different forms of marital status was related to sex, diagnosis year, SEER historic stage, age at diagnosis, AJCC stage, presence or absence of surgery, median household income, and registry sites (*P* < 0.05). Notably, the percentage of widowed patients among the elderly (≥80 years old) was the highest (44.9% vs 3.7%–8.4%, *P* *<* 0.001), while among those less than 40 years old, there tended to be a higher ratio of single patients than in the other three groups (16.3% vs 0%–5.8%, *P* *<* .001). In addition, married patients were more probably to be male (59.1%), and female individuals showed the highest percentage of widowhood (77.4% vs 40.9%–60.2%, *P* *<* 0.001). The widowed group showed a higher prevalence in the SEER metastatic histologic stage than the married patients (2.3% vs 1.0%, *P* *<* 0.026).

### 3.2. Different Types of Marital Status Influenced OS and CSS on UM Patients

**Figure 2:**
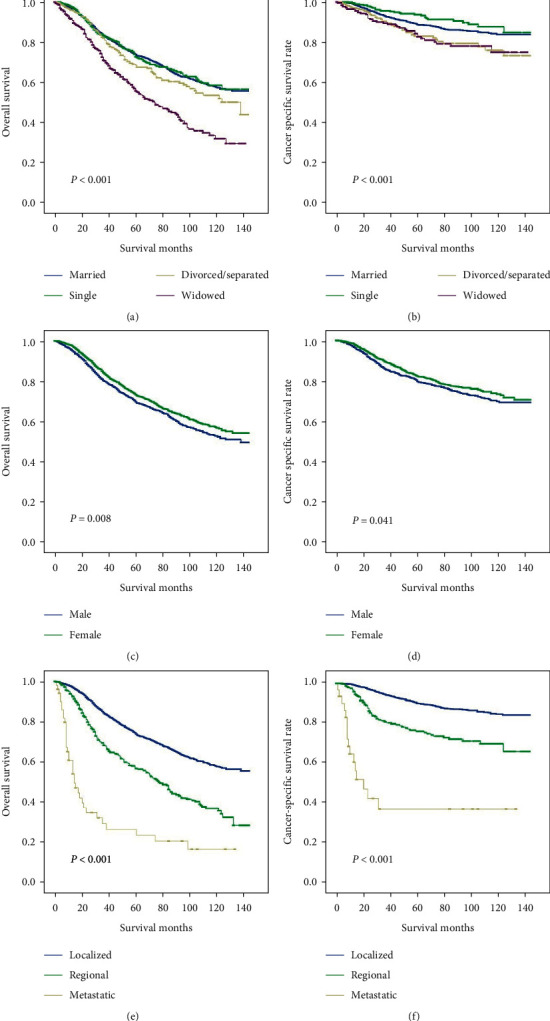
Kaplan–Meier comparison of overall survival (OS) and cancer-specific survival (CSS) (a, b) among patients with married, single, divorced or separated, and widowed status, (c, d) among patients with different genders, and (e, f) among patients stratified by SEER stage.

The results indicated males (*P* = 0.008), elderly (*P* *<* 0.001), black race (*P* = 0.001), widowed marital status (*P* < 0.001), as well as metastatic SEER stage (*P* *<* 0.001) were poor prognostic factors for OS among patients with UM in a univariate analysis. In Figures 2(a) and 2(b), lower OS and CSS occurred in widowed patients in contrast to other groups. Additionally, male and distance stage also showed disadvantages for survival (Figures 2(c)–2(f)). We further conducted multivariate Cox analyses. The results showed that female patients with UM had advantages in OS compared with male patients (HR: 0.770, 95% CI: 0.682–0.870, *P* *<* 0.001). In contrast to the risk in married patients, higher risk of death was found in single individuals (HR: 1.203, 95% CI: 1.005–1.440, *P* = 0.044), divorced/separated group (HR: 1.305, 95% CI: 1.065–1.600, *P* = 0.010), and widowed group (HR: 1.300, 95% CI: 1.086–1.556, *P* = 0.004). Compared with the localized stage group, those in the regional and metastatic stage groups showed noticeably a higher risk of death (HR: 1.985, 95% CI: 1.703–2.313, *P* *<* 0.001; HR: 5.163, 95% CI: 3.681–7.242, *P* *<* 0.001, respectively) (Table 2). The Cox regression analysis of CCS also confirmed the results above. A high mortality rate was linked to patients in the metastatic stage group (HR: 10.733, 95% CI: 6.351–18.141, *P* *<* 0.001). Fewer cancer-related deaths were observed in female compared with male individuals (Table 3).

### 3.3. Subgroup Analyses Stratified by Sex to Investigate Relationship between Marital Status and Survival

**Figure 3:**
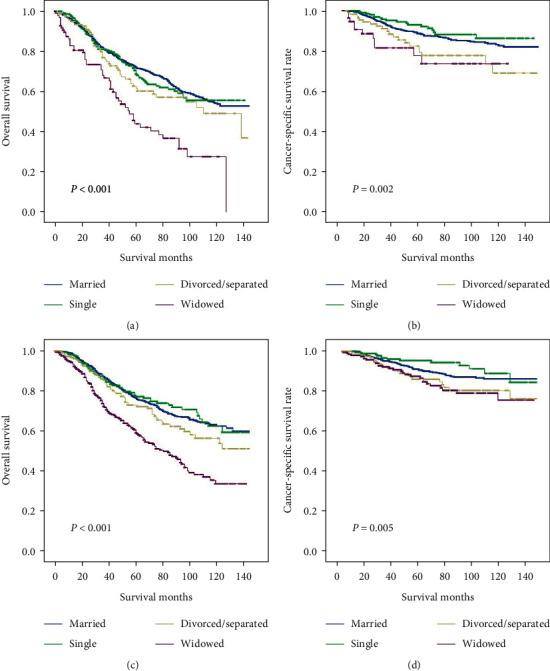
Kaplan–Meier comparison of OS and CSS among patients stratified by gender. (a, b) Male patients and (c, d) female patients.

We explored the impact of marital status on both OS and CCS, which was subgrouped by sex. The sex-specific survival curves of the OS and CCS of four marital statuses are presented in Figure 3. The results of the sex-specific Cox regression are summarized in Tables 4 and 5. In contrast to married patients, widowed patients had a worse prognosis regardless of whether the patients were male or female. The widowed patients had shorter median OS and CCS in the male group (OS: 37 vs 45 months, *P* < 0.001; CSS: 37 vs 47 months, *P* = 0.002) and female group (OS: 42 vs 48 months, *P* < 0.001; CSS: 50 vs 51 months, *P* = 0.005) than others.

In a multivariate analysis, widowed individuals showed notably increased overall death not only in men (HR 2.350, 95% CI 1.764–3.132, *P* < 0.001) but also in women (HR 2.146, 95% CI 1.749–2.633, *P* < 0.001) among four groups. Furthermore, the risk of cancer-specific death in widowed individuals also increased in contrast to married persons, both for males (HR 2.267, 95% CI 1.226–4.193, *P* < 0.001) and females (HR 1.721, 95% CI 1.123–2.637, *P* < 0.001).

### 3.4. Subgroup Analyses Stratified by SEER Stage to Observe the Impact of Marital Status on Survival

**Figure 4:**
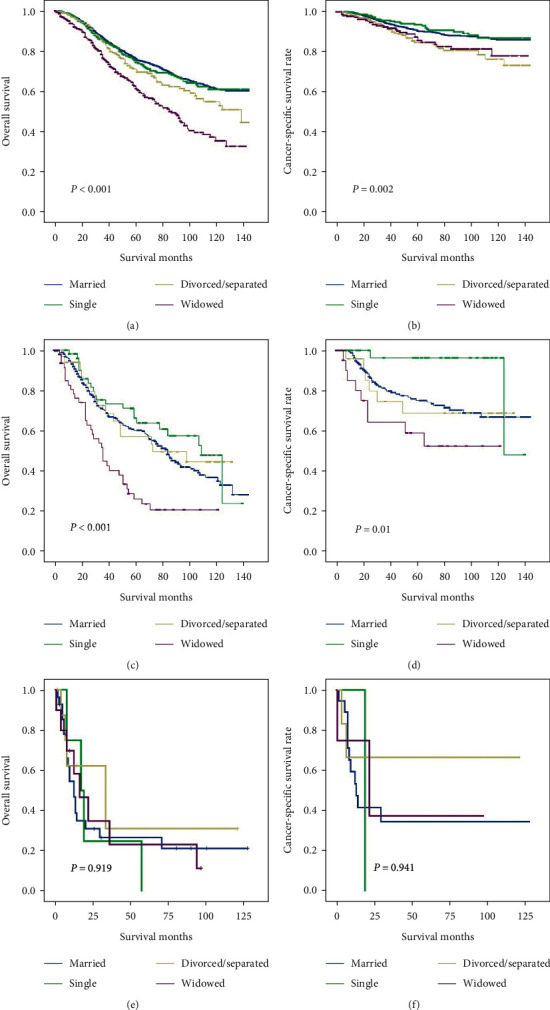
Kaplan–Meier comparison of OS and CSS among patients stratified by SEER stage. (a, b) Patients in the localized stage; (c, d) patients in the regional stage; (e, f) patients in the distant stage.

We then analyzed whether marital status showed any impact UM patients' prognosis, when subgrouped via three SEER stages. The curve and results are shown in Figure 4 and Tables 6 and 7. Compared with the widowed group, the married group had a longer median OS and CCS in the localized stage (OS: 46 vs 43 months, *P* < 0.001; CSS: 49 vs 47.5 months, *P* = 0.002) and regional stage (OS: 54 vs 31 months, *P* < 0.001; CSS: 66.5 vs 37 months, *P* = 0.01) groups. No notable difference was observed between these two groups in the metastatic stage (*P*>0.05).

In a multivariate analysis, widowed patients were observed with obviously increased risk of overall death in comparison with married individuals both in the localized stage (HR 1.953, 95% CI 1.633–2.336, *P* < 0.001) and regional stage (HR 2.161, 95% CI 1.483–3.151, *P* < 0.001) groups. Furthermore, the cancer-specific death in widowed patients was also increased compared with the risk in married patients, both in the localized stage (HR 1.598, 95% CI 1.075–2.374, *P* = 0.02) and regional stage (HR 2.077, 95% CI 1.011–4.268, *P* = 0.047) groups. However, no notable difference was found in OS and CSS in the metastatic group (*P*>0.05).

### 3.5. Subgroup Analyses Stratified by Age at Diagnosis to Study the Influence of Marital Status on Survival

**Figure 5:**
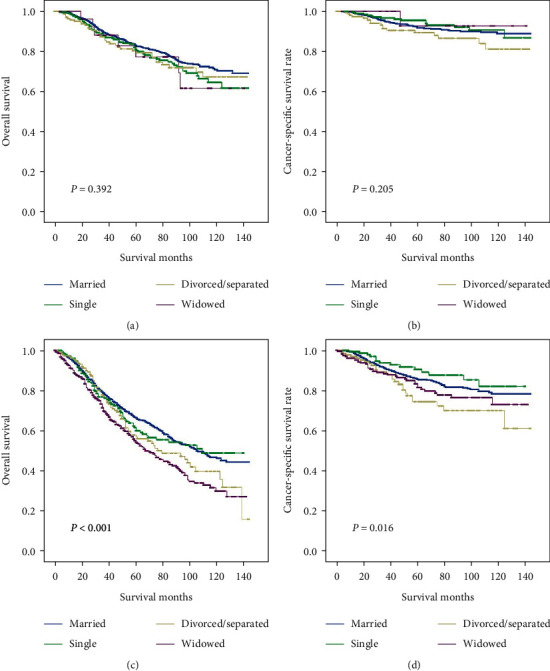
Kaplan–Meier comparison of OS and CSS among patients stratified by age at diagnosis. (a, b) Patients lower than 60 years old and (c, d) patients older than 60 years old.

We divided the UM patients into two groups based on whether over 60 years old. As shown in Figure 5 and Tables 8 and 9, married patients showed better OS and CCS in the older patient group (OS: *χ*^2^ = 25.320, *P* *<* 0.001; CCS: *χ*^2^ = 10.296, *P* *=* 0.016). In the multivariate analysis, patients were observed with notably an increased risk of overall death in comparison with married patients when they were over 60 years old (HR 1.516, 95% CI 1.282–1.791, *P* *<* 0.001). Furthermore, the cancer-specific death in widowed patients was also increased compared with the risk in married patients. In addition, no obvious difference was observed in OS and CSS in patients under 60 years old (*P* > 0.05).

## 4. Discussion

Although the influence of marriage on cancer survival has been investigated in some studies [13–17], no study has emphasized the influence of marital status on UM patients or has conducted subgroup analysis stratified by sex and SEER stage. Here, we sought to investigate the correlations between the marital status and survival outcome in UM patients, who were diagnosed from 2004 to 2015. The results found married patients showed better prognosis than other three groups including single, divorced/separated, and widowed individuals. In particular, widowed individuals, due to some factors, showed lower survival than others.

In present study, the analysis indicated that male sex, elderly age, widowed status, and metastatic histologic stage were closely correlated with the prognosis of UM patients. Subgroup analysis was stratified according to sex, SEER stage, and age. Interestingly, we illuminated the highest mortality rate occurred in widowed individuals not only for male patients but also for females, which was consistent with the aforementioned results. Further subgroup analysis stratified according to the SEER stage demonstrated that married patients in the localized and regional stage groups showed a higher survival rate than widowed patients. There was no significant difference for patients in the metastatic stage group, which might be due to the limited patient numbers in this group. What's more, the results showed the effect of marital status on both OS and CSS in patients over 60 years old. The probable reason why no obvious impact was observed in younger ones was that younger patients could get more support from friends, parents, or other sources, whereas older individuals lived with their spouse mostly.

Research on the impact of psychosocial well-being on survival has yielded various potential mechanisms to explain the relationship between marital status and survival. At first, patients suffer not only physical soreness but also psychological depression. Psychological depression and stress would lead to several dysfunctions, such as hypothalamic-pituitary-adrenal (HPA) axis impairment and activation of inflammatory cytokines [18–20]. The impaired HPA axis results in glucocorticoid resistance and increased catecholamines [21]. Cytokines, including interleukin-6 (IL-6) as well as tumor necrosis factor-*α* (TNF-*α*), mediate interactions with glucocorticoid signaling [22–24]. The disturbance of the HPA axis promotes tumor growth, invasion, and metastasis [25]. Second, unmarried patients, particularly widowed, seemed more probable to live following chronic psychological depression after diagnosis of cancer. In addition, unhealthy lifestyle habits, such as smoking, alcohol, or drug abuse, resulted in poor therapeutic outcomes [26].

The married patients had fewer complications and timelier follow-ups than the other patients during the course of treatment. Some studies reported that the complications noted at follow-up were fewer and that the treatment of married patients was better than those in the other groups. The spouse's support played crucial role in prognosis, due to their encouragement on keeping monitoring and treatment [14, 27]. Magrin et al. conducted a meta-analysis and indicated that mental support from spouses encouraged patients to have better persistence and tolerance with therapy as well as regular follow-up [28]. Moreover, because access to high-quality healthcare is strongly linked to financial status, economic support from the spouse has advantages in terms of improving access to better therapeutic strategies and medications [29, 30]. A recent study found that the differences in economic resources influenced the survival outcomes of cancer patients [29]. Finally, psychosocial and financial support from the spouse may decrease depression and enhance confidence in a patient's ability to triumph over illness [31, 32]. Our analyses were consistent with previously reported studies. Therefore, we suggest that more psychological care and social support could be encouraged to patients as much as possible.

Psychological screening and intervention in the treatment of patients with UM have been recommended [33]. Williamson et al. conducted a 3-month prospective study to investigate the supportive care needed for patients with cancer. The results suggested that effective psychoeducational interventions can be tailored to patients to reduce anxiety as well as address psychological needs and support [33–35]. Therefore, effective and timely psychological care has a beneficial impact on the development of these diseases.

Rajeshuni et al. made evaluation of socioeconomic associations with treatment and survival in UM. The results showed that socioeconomically disadvantaged patients with UM are more likely to be treated with primary enucleation and also revealed opportunities to address issues regarding treatment choice in UM, which was a merit point in this work [36]. However, no study has emphasized the influence of marital status on UM patients or has conducted subgroup analysis stratified by sex and SEER stage. In our present study, we emphasized these points, which was also an merit point in our work.

Although the data we extracted contained multiple UM patients in the United States, there are still some potential limitations in our study that we should consider. First, marital status here was the status when patients were diagnosed. Because the change of marital status after diagnosis was unknown, we cannot investigate the physical and mental care from the spouse on the patient's psychological health. Data on the alterative marital status of cancer patients were not available. Second, the 2010 US Census database indicated that only one-third of unmarried Americans who lived alone were without partners, and the others lived with partners even though their marital status was single [14]. These patients may also enjoy psychosocial and financial support from their partners, which would influence the reliability of our analysis to some extent. Third, this database mainly recorded US population, which could not by totally representative as global populations. Moreover, other important data, such as body mass, diet, and social status, were not included in this study. Therefore, we need to analyze the patients in different countries such as China or other countries and enlarge the samples, which might be more reliable.

## 5. Conclusion

In conclusion, our study indicated that marital status was proved to be an independent prognostic value for survival in UM patients. Better prognosis and therapeutic outcomes were observed in married individuals compared with widowed ones. Greater risks for OS and CSS were observed in unmarried patients, in particular, widowed when compared with married individuals. Hence, we need to provide more physical and psychological care for widowed patients.

## Figures and Tables

**Table 1:** Baseline characteristics of uveal melanoma patients in the SEER database eligible in this study.

Characteristics	Total (%)	Married (%)	Single (%)	Divorced/separated (%)	Widowed (%)	*P*
	4217 (100)	2793 (66.2)	615 (14.6)	379 (9.0)	430 (10.2)	
Gender						<0.001
Male	2215 (52.5)	1652 (59.1)	315 (51.2)	151 (39.8)	97 (22.6)	
Female	2002 (47.5)	1141 (40.9)	300 (48.8)	228 (60.2)	333 (77.4)	
Age at diagnosis						<0.001
18–40	280 (6.6)	162 (5.8)	100 (16.3)	18 (4.7)	0 (0.0)	
40–59	1534 (36.4)	1077 (38.6)	266 (43.3)	162 (42.7)	29 (6.7)	
60–79	1922 (45.6)	1319 (47.2)	210 (34.1)	185 (48.8)	208 (48.4)	
≥80	481 (11.4)	235 (8.4)	39 (6.3)	14 (3.7)	193 (44.9)	
Race						0.907
Black	28 (0.7)	16 (0.6)	6 (1.0)	3 (0.8)	3 (0.7)	
White	4071 (96.5)	2696 (96.5)	589 (95.8)	367 (96.8)	419 (97.4)	
Others	62 (1.5)	42 (1.5)	11 (1.8)	4 (1.1)	5 (1.2)	
Unknown	56 (1.3)	39 (1.4)	9 (1.5)	5 (1.3)	3 (0.7)	
Diagnosis year						0.022
2004–2006	1012 (24.0)	672 (24.1)	137 (22.3)	86 (22.7)	117 (27.2)	
2007–2009	1034 (24.5)	688 (24.6)	133 (21.6)	92 (24.3)	121 (28.1)	
2010–2012	1008 (23.9)	646 (23.1)	168 (27.3)	91 (24.0)	103 (24.0)	
2013–2015	1163 (27.6)	787 (28.2)	177 (28.8)	110 (29.0)	89 (20.7)	
SEER stage						0.026
Localized	3767 (89.3)	2505 (89.7)	556 (90.4)	336 (88.7)	370 (86.0)	
Regional	399 (9.5)	260 (9.3)	55 (8.9)	34 (9.0)	50 (11.6)	
Metastatic	51 (1.2)	28 (1.0)	4 (0.7)	9 (2.4)	10 (2.3)	
Histology type						0.423
Malignant melanoma, NOS	3293 (78.1)	2196 (78.6)	460 (74.8)	301 (79.4)	336 (78.1)	
Mixed epithelioid and spindle cell melanoma	338 (8.0)	212 (7.6)	56 (9.1)	27 (7.1)	43 (10.0)	
Spindle cell melanoma	428 (10.1)	285 (10.2)	71 (11.5)	36 (9.5)	36 (8.4)	
Others	158 (3.7)	100 (3.6)	28 (4.6)	15 (4.0)	15 (3.5)	
AJCC stage						0.037
I	1576 (37.4)	1054 (37.7)	231 (37.6)	146 (38.5)	145 (33.7)	
II	1803 (42.8)	1217 (43, 6)	243 (39.5)	154 (40.6)	189 (44.0)	
III	791 (18.8)	495 (17.7)	137 (22.3)	71 (18.7)	88 (20.5)	
IV	47 (1.1)	27 (1.0)	4 (0.7)	8 (2.1)	8 (1.9)	
Surgery						<0.001
Yes	1232 (29.2)	757 (27.1)	212 (34.5)	128 (33.8)	135 (31.4)	
No/unknown	2985 (70.8)	2036 (72.9)	403 (65.5)	251 (66.2)	295 (68.6)	
Median household income						0.002
Quartile 1	1044 (24.8)	700 (25.1)	112 (18.2)	110 (29.0)	122 (28.4)	
Quartile 2	1057 (25.1)	680 (24.3)	183 (29.8)	88 (23.2)	106 (24.7)	
Quartile 3	1060 (25.1)	720 (25.8)	155 (25.2)	90 (23.7)	95 (22.1)	
Quartile 4	1056 (25.0)	693 (24.8)	165 (26.8)	91 (24.0)	107 (24.9)	
Registry sites						<0.001
West	2322 (55.1)	1534 (54.9)	383 (62.3)	202 (53.3)	203 (47.2)	
Midwest	508 (12.0)	333 (11.9)	63 (10.2)	46 (12.1)	66 (15.3)	
South	802 (19.0)	548 (19.6)	78 (12.7)	85 (22.4)	91 (21.2)	
Northeast	585 (13.9)	378 (13.5)	91 (14.8)	46 (12.1)	70 (16.3)	

**Table 2:** Univariate and multivariate analysis for OS in uveal melanoma patients.

Variables	Median OS (month)	Univariate analysis		Multivariate analysis	
		Log-rank *χ*^2^	*P* value	HR (95% CI)	*P* value
Gender					<0.001
Male	44	7.138	0.008	Reference	
Female	47			0.770 (0.682, 0.870)	
Age at diagnosis		77.138	<0.001		<0.001
<40	51.5			Reference	
40–59	54			1.774 (1.208, 2.604)	0.003
60–79	43			3.136 (2.150, 4.575)	<0.001
≥80	34			7.065 (4.762, 10.482)	<0.001
Race		16.063	0.001		0.001
Black	39			Reference	
White	45			2.307 (0.742, 7.178)	0.149
Others	43.5			2.321 (0.670, 8.042)	0.184
Unknown	74			0.561 (0.125, 2.514)	0.450
Diagnosis year		3.783	0.286		0.058
2004–2006	109				
2007–2009	80				0.025
2010–2012	46				0.023
2013–2015	15				0.856
Marital status		77.138	<0.001		0.003
Married	46			Reference	
Single	44			1.203 (1.005, 1.440)	0.044
Divorced/separated	43			1.305 (1.065, 1.600)	0.010
Widowed	41			1.300 (1.086, 1.556)	0.004
SEER stage		213.756	<0.001		<0.001
Localized	45			Reference	
Regional	50			1.985 (1.703, 2.313)	<0.001
Metastatic	13			5.163 (3.681, 7.242)	<0.001
Surgery		81.276	<0.001		<0.001
Yes	38			Reference	
No/unknown	47			0.588 (0.523, 0.661)	<0.001
Median household income		9.447	0.024		0.145
Quartile 1	45				
Quartile 2	42				0.076
Quartile 3	44.5				0.970
Quartile 4	49				0.046
Registry sites		4.391	0.222		0.015
West	43			Reference	
Midwest	46.5			0.861 (0.715, 1.036)	0.113
South	46			1.056 (0.904, 1.234)	0.492
Northeast	54			0.796 (0.669, 0.947)	0.010

**Table 3:** Univariate and multivariate analysis for CSS in uveal melanoma patients.

Variables	Median CCS (month)	Univariate analysis		Multivariate analysis	
		Log-rank *χ*^2^	*P* value	HR (95% CI)	*P* value
Gender					0.023
Male	47	4.164	0.041	Reference	
Female	50			0.767 (0.610, 0.965)	
Age at diagnosis		21.099	<0.001		<0.001
<40	51.5			Reference	
40–59	57			1.221 (0.680, 2.190)	0.504
60–79	46			2.329 (1.316, 4.124)	0.004
≥80	33.5			3.730 (1.931, 7.205)	<0.001
Race		7.202	0.066		0.158
Black	34			Reference	
White	48			—	0.055
Others	44.5			—	0.673
Unknown	77.5			—	0.031
Diagnosis year		17.289	0.001		0.001
2004–2006	117			Reference	
2007–2009	86			0.588 (0.448, 0.773)	0
2010–2012	48			0.707 (0.522, 0.956)	0.025
2013–2015	15			0.527 (0.324, 0.859)	0.01
Marital status		21.099	<0.001		0.029
Married	49			Reference	
Single	47			0.851 (0.580, 1.247)	0.407
Divorced/separated	46			1.592 (1.139, 2.224)	0.006
Widowed	47			1.254 (0.865, 1.818)	0.233
SEER stage		195.694	<0.001		<0.001
Localized	48			Reference	
Regional	65.5			2.651 (1.989, 3.533)	<0.001
Metastatic	13			10.733 (6.351, 18.141)	<0.001
Surgery		98.593	<0.001		<0.001
Yes	41			Reference	
No/unknown	48			0.456 (0.388, 0.534)	<0.001
Median household income		10.754	0.013		0.320
Quartile 1	47			Reference	
Quartile 2	45			—	0.413
Quartile 3	47			—	0.453
Quartile 4	55			—	0.853
Registry sites		10.613	0.014		0.002
West	46			Reference	
Midwest	50			0.927 (0.639, 1.347)	0.692
South	48			1.620 (1.242, 2.113)	<0.001
Northeast	60			0.923 (0.667, 1.278)	0.631

**Table 4:** Univariate and multivariate analysis for evaluating the influence of marital status on OS according to gender.

Variables	Median OS (month)	Univariate analysis		Multivariate analysis	
		Log-rank *χ*^2^	*P* value	HR (95% CI)	*P* value
Male	44	37.102	<0.001		<0.001
Married	45			Reference	
Single	44			1.053 (0.837, 1.325)	0.658
Divorced/separated	36			1.242 (0.922, 1.672)	0.154
Widowed	37			2.350 (1.764, 3.132)	<0.001
Female	47	61.563	<0.001		<0.001
Married	48			Reference	
Single	44			0.968 (0.732, 1.280)	0.819
Divorced/separated	46.5			1.265 (0.959, 1.670)	0.097
Widowed	42			2.146 (1.749, 2.633)	<0.001

**Table 5:** Univariate and multivariate analysis for evaluating the influence of marital status on CCS according to gender.

Variables	Median CSS (month)	Univariate analysis		Multivariate analysis	
		Log-rank *χ*^2^	*P* value	HR (95% CI)	*P* value
Male	47	14.684	0.002		0.006
Married	47			Reference	
Single	48			0.740 (0.452, 1.210)	0.230
Divorced/separated	40			1.741 (1.089, 2.785)	0.021
Widowed	37			2.267 (1.226, 4.193)	0.009
Female	50	12.744	0.005		0.007
Married	51			Reference	
Single	47			0.675 (0.375, 1.214)	0.189
Divorced/separated	49			1.586 (0.995, 2.527)	0.052
Widowed	50			1.721 (1.123, 2.637)	0.013

**Table 6:** Univariate and multivariate analysis for evaluating the influence of marital status on OS according to SEER stage.

Variables	Median CSS (month)	Univariate analysis		Multivariate analysis	
		Log-rank *χ*2	*P* value	HR (95% CI)	*P* value
Localized		15.140	0.002		0.004
Married	49			Reference	
Single	45			0.848 (0.567, 1.268)	0.422
Divorced/separated	46			1.744 (1.210, 2.516)	0.003
Widowed	47.5			1.598 (1.075, 2.374)	0.020
Regional		11.432	0.010		0.006
Married	66.5			Reference	
Single	77			0.209 (0.051, 0.861)	0.030
Divorced/separated	49			1.024 (0.435, 2.407)	0.957
Widowed	37			2.077 (1.011, 4.268)	0.047
Metastatic		0.397	0.941		0.943
Married	13			Reference	
Single	20			—	0.798
Divorced/separated	9			—	0.613
Widowed	16.5			—	0.831

**Table 7:** Univariate and multivariate analysis for evaluating the influence of marital status on CCS according to SEER stage.

Variables	Median OS (month)	Univariate analysis		Multivariate analysis	
		Log-rank *χ*^2^	*P* value	HR (95% CI)	*P* value
Localized		57.760	<0.001		<0.001
Married	46			Reference	
Single	43			1.051 (0.864, 1.278)	0.620
Divorced/separated	44			1.260 (1.010, 1.573)	0.041
Widowed	43			1.953 (1.633, 2.336)	<0.001
Regional		22.116	<0.001		<0.001
Married	54			Reference	
Single	60			0.751 (0.478, 1.179)	0.213
Divorced/separated	46.5			0.875 (0.513, 1.494)	0.625
Widowed	31			2.161 (1.483, 3.151)	<0.001
Metastatic		0.500	0.919		0.924
Married	11.5			Reference	
Single	19			—	0.877
Divorced/separated	9			—	0.526
Widowed	15			—	0.880

**Table 8:** Multivariate analysis for evaluating the influence of marital status on OS according to age.

Variables	Median OS (month)	Univariate analysis		Multivariate analysis	
		Log-rank *χ*2	*P* value	HR (95% CI)	*P* value
<60	54	2.998	0.392		0.395
Married				Reference	
Single				—	0.158
Divorced/separated				—	0.283
Widowed				—	0.467
≥60	40	25.320	<0.001		<0.001
Married				Reference	
Single				1.060 (0.836, 1.344)	0.628
Divorced/separated				1.248 (0.978, 1.593)	0.076
Widowed				1.516 (1.282, 1.791)	<0.001

**Table 9:** Multivariate analysis for evaluating the influence of marital status on CSS according to age.

Variables	Median CSS (month)	Univariate analysis		Multivariate analysis	
		Log-rank *χ*^2^	*P* value	HR (95% CI)	*P* value
<60	59	4.585	0.205		0.215
Married				Reference	
Single				—	0.468
Divorced/separated				—	0.082
Widowed				—	0.606
≥60	46	10.296	0.016		0.021
Married				Reference	
Single				0.715 (0.420, 1.219)	0.218
Divorced/separated				1.673 (1.100, 2.543)	0.016
Widowed				1.333 (0.934, 1.901)	0.113

## Data Availability

The data used to support the findings of this study are included within the article.
